# Supplementary material for: Protective efficacy of six recombinant proteins as vaccine candidates against Echinococcus granulosus in dogs
Source: PLoS Negl Trop Dis. 2023 Oct 23;17(10):e0011709. doi: 10.1371/journal.pntd.0011709 (PMC10621941; doi:10.1371/journal.pntd.0011709)
Supplement: Attachment — Submitted filename: Point-by-point response to reviewers and editors-revised.docx [file pntd.0011709.s002.docx]

Dear Reviewers and Editors,

On behalf of all the co-authors, we extend our sincere gratitude to you and the esteemed reviewers for providing insightful comments and invaluable suggestions regarding our manuscript "Protective efficacy of six recombinant proteins as vaccine candidates against *Echinococcus granulosus* in dogs". The peer reviewers considered that our manuscript is of interest to the readership of PLOS Neglected Tropical Diseases and would be worthy of publication after minor revisions. The reviewers' discerning feedback has guided us in improving this article. In accordance with the reviewers' comments and suggestions, we have meticulously revised the manuscript. The detailed point-by-point responses to all review comments are provided below for your reference, and we have highlighted the revised text in the manuscript. Notably, we are glad to make the review history publicly available.

We sincerely hope that the manuscript has been revised to your satisfaction and look forward to hearing its acceptance for publication in PLOS Neglected Tropical Diseases. Thank you very much again for your attention and consideration of our manuscript.

Sincerely yours,

Guangyou Yang, PhD

Professor, Department of Parasitology,

College of Veterinary Medicine, Sichuan Agricultural University,

211 Huiming Road, Wenjiang, 611130, Sichuan Province, China

Tel: 086-028-86291162; Fax: 086-028-86292116

E-mail: [guangyou1963@126.com](mailto:guangyou1963@126.com)

**Responses to comments and suggestions of Reviewer 1:**

**General comments and suggestions:**

It is a paper that can mark an important change in CE control

We understand that the introduction can be improved by ordering the different possible interventions. Include more background. For example, there are programs where the PZQ has been successful in decreasing the number of human cases. The problems pointed out by the authors are correct. Secondly, vaccination in sheep also presents a history of success in stopping transmission. Also, the problems mentioned regarding the size of the population to be vaccinated are correct. So, it would be better to present vaccination in dogs as a new alternative that in principle can complement the previous ones. I would avoid using the term eradication. It is better to talk about eliminating the transmission to man. The experiences of use of EG95 include 2 doses and a booster per year. or more. See papers of Rio Negro or Morocco.

**Response:** On behalf of all the contributing authors, we would like to express our sincere appreciations of your constructive comments concerning our article. These comments are all valuable and helpful for improving our article. As suggested, we have substituted the word “eradication” with “eliminate the transmission of E. granulosus to human and livestock” (line 100-102). Moreover, we have added more detailed information to the use of EG95 in the manuscript (Line 92-95; 115-118).

**Minor points**

**Point 1:** in results, can the authors indicate the exact values of P?

**Response:** As suggested, we have indicated the exact values of *P* in results (line 211-217).

**Point 2:** There should be a mention that the duration of the immune response has not yet been determined.

**Response:** We thank for constructive suggestions on our manuscript. To enhance clarity, we have added this statement in line 224-225.

**Responses to comments and suggestions of Reviewer 2:**

**General comments and suggestions:**

The submitted article named “Protective efficacy of six recombinant proteins as vaccine candidates against Echinococcus granulosus in dogs” is a well written article that explores the use of three vaccine formulations (each composed of two recombinant antigens) to limit the establishment of Echinococcus granulosus sensu lato infection in dogs. As dogs plays an important role in the E. granulosus life cycle as the definitive host, a successful vaccine could indirectly protect both farm animals and humans, as both act as intermediate hosts. Moreover, as there are a lot fewer dogs than farm animals in rural populations, dogs vaccination programs are usually consider as a more cost- and time-effective strategy.

This article is easy to read, well written and self-explanatory. The methods are consistent with the objectives, and the obtained results support the conclusion.

There are some key comments I would like to point out

**Point 1:** E. granulosus s.l. genomic differences:

E. granulosus is a complex of parasites divided into nine genotypes grouped in four species. There are significant genomic differences between them, and not all genotypes are fully sequenced. It would be advice to genotype the protoscoleces used for infections / obtained worms (or at least do a bibliographical research of the genotypes present on sheeps in China) and evaluate whether the recombinant antigens have any amino acid sequence difference between the canonical gen (G1) and the genotypes present. Not knowing this could be playing against you, as the dogs’ infections might be with G1 and G5, for example, and the vaccine only protect against G1.

**Response:** We sincerely thank for your valuable feedback that we have used to improve the quality of our manuscript. We have thoroughly reviewed your considerations on the genotypes of the protoscoleces used for infections in this study. In Materials and methods section, we have provided the bibliographical research of the genotypes present in China (line 146) and genotyped the PSCs as *E*. *granulosus* sensu stricto (G1-G3) used in this study (line 142-147). Moreover, the amino acid sequences of recombinant antigens are the same with *E*. *granulosus* G1 genotype.

**Point 2:** Measurement of mucosal immunity

The consensus when developing a vaccine against an intestine parasite is that a significant mucosal immunity should be achieved. In fact, the salmonella vaccine using A31-Trp has shown that this response is greatly protective. It is known that oral vaccines can generate better mucosal immunity. Why was the selection of the immunization scheme subcutaneous, and why was not a measurement of serum/mucosal IgAs.

**Response:** We feel great thanks for your professional questions.

We chose the subcutaneous immunization scheme based on the success of previous E. granulosus vaccines for dogs as we mentioned in the Introduction section (lines 110-112), which adopted a subcutaneous injection and proved effective. Additionally, your insightful inquiries of an oral vaccine have provided a promising direction for our future research.

Regarding your question about measuring serum/mucosal IgAs, we acknowledge that this is a limitation in our study. Our experiments primarily focused on evaluating humoral immunity and did not detect the mucosal immune response. We appreciate your suggestion, and we will consider incorporating improvements into our following experimental designs for assessing immune protection.

**Point 3:** Other considerations

When describing the vaccine protocol in dogs, it should be mentioned that weekly sera extractions were performed for further IgG quantification.

**Response:** As suggested, we have added the information at line 180-181.

**Minor points**
**Point 1:** I would like to recommend some text modifications to improve the overall reading experience: First of all, when the recombinant antigens are first introduced, I would explicitly say the whole name of the protein (eg. EgANXB3: Annexin B3) as it might help to do parallelism with other pathogen vaccines.

**Response:** We thank for constructive suggestions on our manuscript. To enhance clarity, we have made revisions in the manuscript (line 37-40).

**Point 2:** Secondly, when mentioning the current dog vaccine (line 111), the composition of the available vaccine/vaccines should be mentioned, as its not clear if it is a crude antigen vaccine, recombinant vaccine or purified vaccine. This also makes the “cost” referred in line 113 not easily associated to the vaccine development, as a crude antigen vaccine industrial processes in unthinkable due to E. granulosus not being a cultivable parasite. Also, there should be more context around EG95.

**Response:** We feel sorry for confusing the readers. To enhance clarity, we have added the detailed vaccine information as “recombinant vaccine” in the manuscript. Moreover, we have provided more detailed information around EG95 (line 92-95; 115-118).

**Point 3:** On the Salmonella A31-Trp vaccine, they already use a combination of two antigens, making your overall innovating two-protein strategy, although interesting, not fully innovating

Then describing the “two-protein combined vaccine administration” it should reflect that there are two different system applied, one with the co-administration of two proteins separately, and one with the administration of two fused proteins.

**Response:** Thank you for your reminder. We have re-written this part to make it clarify (line 126-130).

**Point 4:** It would be advice to compare the worm reduction values here obtained with the previously reported

**Response:** We thank for constructive suggestions on our manuscript. We have added the detailed data information of worm reduction values as suggested for comparison (line 270-273).

**Point 5:** On line 266, Salmonella A31-Trp vaccine did not have any associated risk, the genetically modified salmonella cannot regain their pathogenesis mechanisms, and the biodistribution of salmonella was previously analyzed, and shown to be safe. The biological risk that is mentioned is the possibility of developed worms (in either control or vaccinated dogs) to produce oncospheres that may infect the handlers, which is also present in this article

**Response:** We sincerely thank you for careful reading. As suggested, we have re-written our statement on line 279-280.

**Point 6:** On line 239 and 277, the “rEgTIM-rEgANXB3” should not include the “-“ as both antigens are not conjugated, but co-administrated

**Response:** We feel sorry for our carelessness. We have made revisions in the manuscript on line 252 and 291.

**Point 7:** Quil-A® is a registered trademark and should include the ®

**Response:** As suggested, we have added this information.

**Responses to comments and suggestions of Reviewer 3:**

**General comments and suggestions:**

Congratulations to the authors for this interesting work, especially as this is a recombinant vaccine trial based on a combination of two proteins at the same time, knowing that dog vaccination is a very important element in the control of this zoonosis.

The comments made do not undermine the quality of this work.

**Point 1:** Line 130: The dogs were treated with Albendazol and Prziquantel, with no residual effect. Have you taken fecal samples to ensure that the dogs are free of E. granulosus parasites or eggs?

**Response:** We feel great thanks for your professional review work on our article. As you are concerned, the half-life time albendazole is 8-12 hours in humans and other species (Dollery, 1999b; EMEA, 1997), and praziquantel are completely cleared within 4 days after an oral dose (Dollery, 1999a). In this study, all the dogs were dewormed with albendazole and praziquantel half month prior to the primary vaccination. Theocratically, there is no residual effect.

Moreover, all dogs were housed in Specific Pathogen Free conditions and subjected to rigorous examination utilizing the purging method to ensure the absence of parasite infections.

**Point 2:** Line 139: On what basis did you select the proteins used in the vaccine? Why these sequences and not others?

**Response:** We feel great thanks for your questions on our article. We select these proteins based on our previous studies, these proteins have been demonstrated to play a crucial role in the development and survival of *E*. *granulosus*, and some of them were assumed to be potential vaccine candidates in previous reports. Thus, we have chosen these proteins. Moreover, we have discussed this question in the line 233-236.

**Point 3:** Line 169: After the intestinal scraping, especially in the control dogs, did you find any eggs in the adult parasite worms collected?

**Response:** Thanks for your questions. We did not find any eggs in the collected worms. For *E*. *granulosus*, the maturity of adult worms with eggs occurs in the dog small intestine at least 32 days of the ingestion of viable protoscoleces. In this study, all dogs were humanely euthanized and underwent necropsy at 28 days to avoid the potential biological risk.

**Point 4:** Line 171: in table 1, why did you use a 200µg dose of the EgFABP1-EgA31 vaccine and not a 100µg dose like the others?

**Response:** We sincerely thank you for careful reading. We have introduced an approach that involves the development of a two-protein combined vaccine which applied two different systems, one with the co-administration of two proteins (100µg /protein/dose) separately, and one with the administration of a fused protein of EgFABP1-EgA31 (200µg /dose). We have added this information in line 126-130 to make our statements clarify.

**Point 5:** Line 220: On what basis did you combine the different proteins?

**Response:** Thanks for your questions. We combined different proteins based on previous studies of parasite vaccines which can enhance the protection stability using two antigen components (Lightowlers, Colebrook et al. 2003). Meanwhile, we based on these proteins’ crucial function in the development and survival of *E*. *granulosus* to make reasonable hypothesis for combination of different proteins. This part has been discussed in the line 233-254.
